# Complete nitrification: insights into the ecophysiology of comammox *Nitrospira*

**DOI:** 10.1007/s00253-018-9486-3

**Published:** 2018-11-10

**Authors:** Hanna Koch, Maartje A. H. J. van Kessel, Sebastian Lücker

**Affiliations:** Department of Microbiology, Radboud University Nijmegen, Nijmegen, The Netherlands

**Keywords:** Nitrification, Comammox, *Nitrospira*, Complete nitrification, Nitrite-oxidizing bacteria, Ammonia-oxidizing microorganisms

## Abstract

Nitrification, the oxidation of ammonia via nitrite to nitrate, has been considered to be a stepwise process mediated by two distinct functional groups of microorganisms. The identification of complete nitrifying *Nitrospira* challenged not only the paradigm of labor division in nitrification, it also raises fundamental questions regarding the environmental distribution, diversity, and ecological significance of complete nitrifiers compared to canonical nitrifying microorganisms. Recent genomic and physiological surveys identified factors controlling their ecology and niche specialization, which thus potentially regulate abundances and population dynamics of the different nitrifying guilds. This review summarizes the recently obtained insights into metabolic differences of the known nitrifiers and discusses these in light of potential functional adaptation and niche differentiation between canonical and complete nitrifiers.

**Electronic supplementary material:**

The online version of this article (10.1007/s00253-018-9486-3) contains supplementary material, which is available to authorized users.

## Introduction

Nitrification, the sequential aerobic oxidation of ammonia to nitrate via nitrite, is a central nitrogen (N) cycling process. On the one hand, nitrification depletes the pool of accessible ammonium, the best accessible N source for biomass production. On the other hand, the products of nitrification nitrite and nitrate are widely used electron acceptors. From an anthropogenic perspective, nitrification has starkly contrasting roles. It contributes to N loss from fertilized agricultural soils by producing nitrite and nitrate, two compounds that are rapidly reduced to N-containing gases, including the potent greenhouse gas nitrous oxide (N_2_O). Additionally, nitrite and nitrate can be easily washed out from the soil matrix, and thus leach into the groundwater and aquatic ecosystems. The increased N availability in these systems causes a boost of productivity with tremendous consequences, including eutrophication of rivers and lakes, algal blooms, and formation of dead zones in coastal regions. In contrast, nitrification represents the initial N cycling step in biological wastewater treatment, where well-orchestrated microbial activities result in the removal of excess N compounds.

Since the first discovery of nitrifying microorganisms by Sergei Winogradsky at the end of the nineteenth century (Winogradsky 1891), it was believed that nitrification is a two-step process performed by two distinct functional groups, the ammonia- and nitrite-oxidizing bacteria (AOB and NOB, respectively). However, the development of molecular techniques and novel isolation approaches tremendously improved our knowledge of the environmental key players performing this process. A milestone in nitrification research was the discovery of autotrophic ammonia-oxidizing archaea (AOA). Shortly after first metagenomic indications of archaea possessing the genetic inventory for ammonia oxidation, the marine AOA *Nitrosopumilus maritimus* was successfully isolated (Könneke et al. 2005; Treusch et al. 2005). Since the identification of archaeal ammonia oxidizers, numerous studies have focused on their environmental distribution, physiology, and genomics to elucidate their ecological significance and potential factors for niche differentiation between AOA and their bacterial counterpart (reviewed in, e.g., Hatzenpichler 2012; Prosser and Nicol 2012).

In 2015, the surprising identification of microorganisms preforming complete nitrification on their own challenged the strict division of labor between the two nitrifying guilds, and thus caused another paradigm shift in our understanding of nitrification (Daims et al. 2015; van Kessel et al. 2015). Notably, earlier theoretical studies already discussed the existence and possible niches of comammox (COMplete AMMonium OXidation) microorganisms (Costa et al. 2006; van de Leemput et al. 2011). It was hypothesized that the truncation of nitrification might reduce the metabolic cost for a cell compared to performing the whole pathway, resulting in higher growth rates but lower yields. However, a high-growth yield as postulated for comammox organisms might be advantageous in nutrient-limited, slow growth-favoring systems with low-cell washout rates, as for instance found in biofilms. Indeed, the first comammox enrichment cultures were obtained from biofilm samples (Daims et al. 2015; van Kessel et al. 2015). Surprisingly, when analyzing the metagenomes of these enrichment cultures both research groups identified the gene set for complete nitrification in genome bins assigned to *Nitrospira*. Members of the genus *Nitrospira* have been identified as key NOB in diverse natural and man-made systems (Daebeler et al. 2014; Daims et al. 2001; Feng et al. 2016), but were assumed to comprise only autotrophic nitrite oxidizers. All known comammox *Nitrospira* belong to lineage II, the environmentally most widespread clade of this diverse genus, which can be phylogenetically divided into at least six lineages (Daims et al. 2016). Based on phylogenetic analyses of subunit A of the ammonia monooxygenase (AMO), the enzyme that oxidizes ammonia to hydroxylamine, comammox bacteria can be further separated into two monophyletic sister clades, designated clades A and B (Daims et al. 2015). All described comammox cultures obtained so far contain members of clade A and have been enriched from man-made systems, including a biofiltration unit of a recirculation aquaculture system (RAS; *Ca*. N. nitrosa and *Ca*. N. nitrificans; van Kessel et al. 2015) and a biofilm sustained in thermal waters from a 1200-m deep oil exploration well (*N. inopinata*; Daims et al. 2015). In addition, analyzing metagenome-assembled genomes (MAGs) of clade B comammox *Nitrospira* gave first genomic insights into this group so far missing a cultured representative (Orellana et al. 2018; Palomo et al. 2018). Since the discovery of complete nitrifying *Nitrospira*, numerous studies have addressed their environmental distribution and abundance (e.g., Bartelme et al. 2017; Fowler et al. 2018; Hu and He 2017; Orellana et al. 2018; Pjevac et al. 2017) as well as their potential metabolic capabilities by (meta) genomic analyses (e.g., Camejo et al. 2017; Orellana et al. 2018; Palomo et al. 2016; Palomo et al. 2018; Wang et al. 2017). Additionally, physiological investigations of the first comammox pure culture revealed vital insights into the nitrification kinetics of complete compared to canonical nitrifiers (Kits et al. 2017). Recent review papers focused on microbial driven N cycling processes (Kuypers et al. 2018), the enzymatic aspects in nitrification (Lancaster et al. 2018), ammonia oxidation in soil (Beeckman et al. 2018), alternative roles of *Nitrospira* beyond nitrite oxidation (Daims et al. 2016), the biotechnological potential of the comammox process (Lawson and Lücker 2018), and summarized the published literature on comammox organisms (Hu and He 2017). In this review, we cover the main metabolic differences potentially driving niche specialization between comammox *Nitrospira* and canonical ammonia and nitrite oxidizers.

## Environmental distribution of comammox *Nitrospira* compared to other nitrifying guilds

**Fig. 1:**
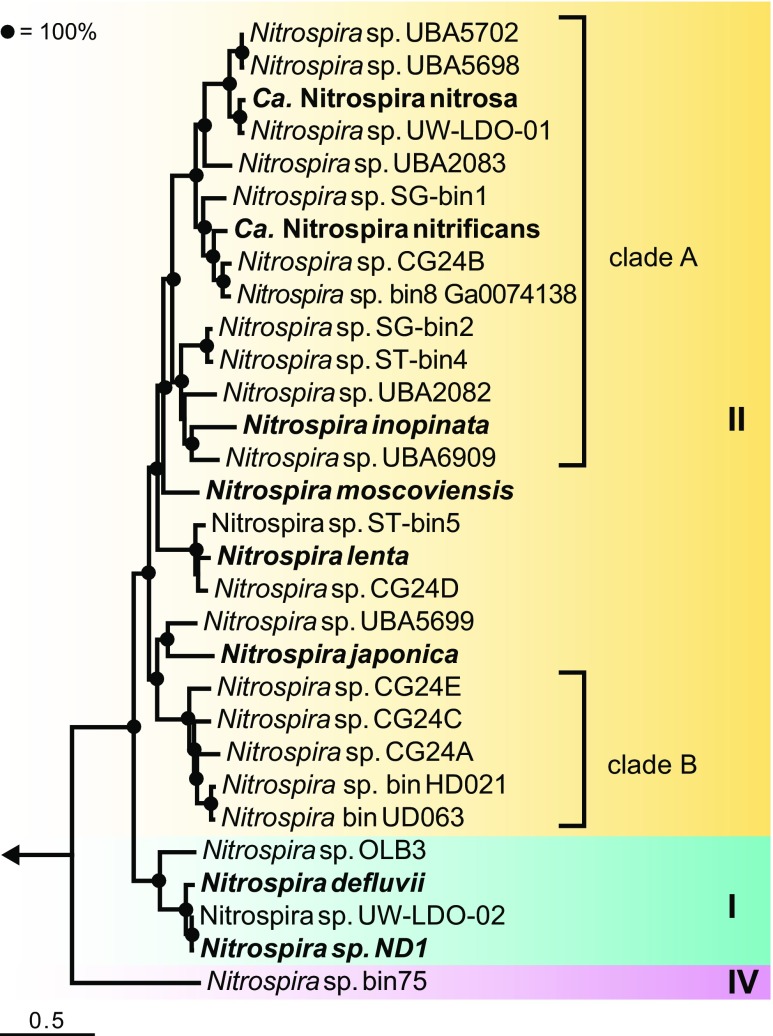
Phylogenetic analysis of the genus *Nitrospira* based on 91 core genes. The UBCG pipeline was used to identify the core gene set consisting of single-copy genes found in most bacterial genomes and for the concatenation of the nucleotide sequence alignments (Na et al. 2018). The tree was reconstructed using RaxML (Stamatakis 2014) on the CIPRES Science Gateway (Miller et al. 2010), using the GTR substitution and GAMMA rate heterogeneity models and 100 bootstrap iterations. *Nitrospira* lineages are indicated by colored boxes and labeled with roman numerals, comammox clades are designated by square brackets. Two *Leptospirillum* species were included into the analysis and used for rooting the tree. The position of the outgroup is indicated by the arrow. The scale bar corresponds to 50% estimated sequence divergence. Only genomes with a predicted completeness of > 85% were included in the phylogenetic analysis. For details, see Table S1

The discovery of complete nitrifiers raises questions about (i) the ecological significance of the comammox process, (ii) driving factors for niche separation between the different ammonia-oxidizing guilds, and (iii) the physiology of comammox compared to strict nitrite-oxidizing *Nitrospira*. Comparing the distribution and abundance of complete nitrifiers to other ammonia oxidizers is a first step towards determining the contribution of the comammox process to nitrification in different environments. However, since comammox bacteria do not form a monophyletic group within *Nitrospira* lineage II (Fig. 1), comammox and canonical nitrite-oxidizing *Nitrospira* cannot be distinguished by 16S rRNA-based methods (Pjevac et al. 2017). Thus, other molecular techniques such as metagenomics and functional gene-based PCR assays have been used to detect complete nitrifiers in environmental samples. In addition to these already applied methods, comammox *Nitrospira* might be visualized in situ using direct-geneFISH (Barrero-Canosa et al. 2017) to detect the *amoA* gene, which encodes subunit A of the AMO, or by immunofluorescence targeting the AMO protein, like performed for AOB (Fiencke and Bock 2004). MAGs assigned to comammox *Nitrospira* have been identified mainly in metagenomes derived from engineered systems, but also from natural ecosystems like fertilized soil (Table S1). For PCR-based approaches, a widely used functional marker of aerobic ammonia oxidation is the *amoA* gene. Recently, several different PCR assays and primer sets targeting comammox *amoA* genes were developed (Bartelme et al. 2017; Fowler et al. 2018; Pjevac et al. 2017; Wang et al. 2017). Although the two-step PCR approach of Wang and co-workers is a valuable tool to identify potential novel members of the copper containing membrane monooxygenase family (Wang et al. 2017), other newly developed comammox *amoA*-targeting qPCR approaches with near-complete group coverages are more suitable to analyze comammox distribution and abundance in environmental samples (Fowler et al. 2018; Pjevac et al. 2017). By applying PCR assays that target comammox clade A and B *amoA* separately, complete nitrifiers could be detected in a wide range of environmental samples, including man-made systems like drinking and wastewater treatment plants, and several natural habitats, like forest and paddy field soils, rice rhizosphere, and lake sediments (Pjevac et al. 2017). In addition to environmental distribution studies, the relative abundance of comammox bacteria compared to canonical ammonia oxidizers has been explored in several ecosystems to elucidate their potential contribution to nitrification. Although large-scale surveys comparing the abundances of ammonia-oxidizing guilds are still missing, first quantitative studies showed co-occurrence of all three ammonia-oxidizing guilds with varying abundance patterns in different habitats (Bartelme et al. 2017; Fowler et al. 2018; Pjevac et al. 2017; Orellana et al. 2018). In engineered systems, like RAS biofilters and groundwater-fed rapid sand filters, comammox *Nitrospira* outnumbered AOB and AOA (Bartelme et al. 2017; Fowler et al. 2018). Notably, timecourse analysis showed that AOA and comammox *Nitrospira* stably co-existed in a RAS biofilter microbial community (Bartelme et al. 2017). One potential factor for the high abundance of comammox *Nitrospira* compared to canonical ammonia oxidizers in these engineered environments might be that the operational setups of these systems favor surface-attached microbial communities, in which complete nitrifiers might benefit from their higher growth yield (Costa et al. 2006; Kits et al. 2017). In addition, the assignment of more than half of the bacterial *amoA* reads in fertilized soil metagenomes to *Nitrospira* suggests a high abundance of complete nitrifiers in natural ecosystems with elevated N inputs (Orellana et al. 2018).

## Potential niche-defining differences between strict ammonia oxidizers and complete nitrifiers

Based on relative abundance measures, comammox *Nitrospira* rarely is the only nitrifying guild present in a habitat. This co-occurrence of comammox and canonical ammonia oxidizers indicates a potential functional differentiation between these microbial groups (Annavajhala et al. 2018; Bartelme et al. 2017; Fowler et al. 2018; Orellana et al. 2018; Palomo et al. 2018; Pjevac et al. 2017). While for AOA and AOB the main physiological factors for niche separation were considered to be mixotrophy, ammonia affinities, and different pH optima (Prosser and Nicol 2012), little is known about potential factors driving niche specialization between comammox and canonical ammonia oxidizers.

**Fig. 2:**
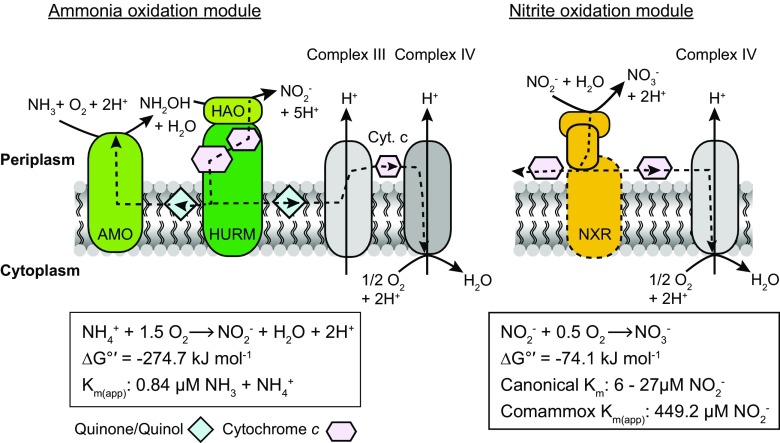
Schematic representation of the ammonia and nitrite oxidation modules in *Nitrospira*, including their incorporation into the respiratory chain for energy conservation. The overall reactions, their standard free energies, and the apparent substrate affinities for comammox and canonical *Nitrospira* are indicated below the figure. AMO, ammonia monooxygenase; HAO, hydroxylamine dehydrogenase; HURM, hydroxylamine-ubiquinone reaction module; NXR, nitrite oxidoreductase; Cyt. c, cytochrome *c*. The complexes of the respiratory chain are indicated by roman numerals. Stippled arrows indicate electron flow. NO as putative intermediate of NH_2_OH oxidation is not shown for simplicity. For details on the modules, see main text. Ammonia and nitrite *K*_*M*_ values were taken from Kits et al. (2017), Nowka et al. (2015a), and Ushiki et al. (2017)

The current knowledge on aerobic ammonia oxidation has been recently summarized (Beeckman et al. 2018; Lancaster et al. 2018). Briefly, key enzymes of bacterial ammonia oxidation include the membrane-associated AMO and the periplasmic hydroxylamine dehydrogenase (HAO), which together with the cytochromes *c*554 and *c*_M_552 forms the hydroxylamine-ubiquinone redox module (HURM; Klotz and Stein 2008). All genes for ammonia and hydroxylamine oxidation have been identified in comammox *Nitrospira* (Fig. 2) and are most similar to betaproteobacterial AOB, indicating an evolutionary link between the ammonia oxidation machineries of these phylogenetically distinct groups (Daims et al. 2015; Palomo et al. 2018; van Kessel et al. 2015). In aerobic ammonia oxidizers, the three-subunit enzyme AMO (encoded by *amoCAB*) initiates nitrification by oxidizing ammonia to hydroxylamine, a reaction that requires molecular oxygen for the activation of ammonia. In AOB, the intermediate hydroxylamine (NH_2_OH) is further oxidized by HAO, while AOA apparently lack a HAO homolog. Intriguingly, recent biochemical investigations of the purified HAO of *Nitrosomonas europaea* suggested that the product of hydroxylamine oxidation might be nitric oxide (NO) and not nitrite as assumed previously (Caranto and Lancaster 2017). This “NH_2_OH/NO obligate intermediate” model proposes the need of a third enzymatic partner of AMO and HAO for bacterial ammonia oxidation to nitrite. The shared sequence similarity of AMO and HAO in AOB and comammox points to comparable ammonia oxidation mechanisms including NO as an obligate intermediate in these nitrifying guilds. Notably, recent investigations of the nitrification kinetics of the pure culture *N. inopinata* revealed a higher apparent ammonia affinity for this comammox bacterium compared to canonical AOB (Kits et al. 2017). Intriguingly, the affinity of *N. inopinata* was found to be even higher than those of most terrestrial AOA, which were previously assumed to drive ammonia oxidation under low substrate concentrations based on the low *K*_*M*_ for ammonia of the marine AOA *N. maritimus* (Martens-Habbena et al. 2009). Moreover, comparative genomic studies identified differences in copy numbers and genomic arrangement of the ammonia oxidation machineries in the nitrifying guilds. Betaproteobacterial AOB possess up to three copies of *haoAB*-*cycAB* encoding the HURM complex, and one to two copies of the *amoCABDE* genes for the AMO holoenzyme and two periplasmic membrane-associated proteins, potentially involved in electron transport (El Sheikh et al. 2008; Kozlowski et al. 2016a). In contrast, comammox *Nitrospira* genomes contain one single gene cluster harboring all *amo* and *hao* genes. The only exceptions are *Ca*. N. nitrosa and *Ca*. N. nitrificans, which possess duplicated *amoA* or *haoA* genes, respectively, and *N. inopinata* where the AMO and HURM gene clusters are separated (Daims et al. 2015; Palomo et al. 2018; van Kessel et al. 2015). In addition, genes for the type I cytochrome *c* biosynthesis are located in this gene cluster. All genomes of betaproteobacterial AOB as well as comammox *Nitrospira* contain at least one additional, non-operonal *amoC* gene. In AOB, this singleton AmoC may be involved in the response to cellular stress, like starvation and elevated temperatures, since the *amoC*_3_ gene is under regulation of the global stress response regulator σ^32^ (Berube and Stahl 2012). Unique features of the comammox ammonia oxidation machinery gene cluster, like the co-localization of AMO, HAO, and cytochrome *c* biosynthesis genes and a duplication of *amoD*, suggest a common origin of this genomic region in comammox clade A and clade B (Palomo et al. 2018). Interestingly, in contrast to the distinct forms of AMO, phylogenetic analyses of HaoA showed no clear separation of the two comammox clades (Fig. S1), indicating a horizontal transfer of HAO between complete nitrifiers of different clades (Palomo et al. 2018). This, together with the separate branching of clade A and B within lineage II (Fig. 1) indicates a complex evolutionary history of comammox *Nitrospira*.

Besides key characteristics of the ammonia-oxidizing machinery, like high ammonia affinity and low maximum ammonia oxidation rate, potential niche separating physiological characteristics include the high growth yield of *N. inopinata* compared to other aerobic ammonia oxidizers (Kits et al. 2017). One metabolic feature influencing the growth yield is the pathway used for carbon fixation, which differs between the nitrifying guilds. AOA fix CO_2_ by using a unique, highly energy efficient variant of the 3-hydroxypropionate/4-hydroxybutyrate pathway, while AOB use the energy-demanding Calvin-Benson-Bassham cycle (Könneke et al. 2014). In contrast, *Nitrospira* fix CO_2_ via the reductive tricarboxylic acid (rTCA) cycle (Lücker et al. 2010), which is found mainly in anaerobic and microaerophilic bacteria due to the O_2_ sensitivity of the key enzymes 2-oxoglutarate:ferredoxin oxidoreductase and pyruvate:ferredoxin oxidoreductase (Campbell et al. 2006). This O_2_ sensitivity might be reduced by the use of the five-subunit isoforms of these enzymes found to be conserved in *Nitrospira*, which have been shown to be functional under oxic conditions in *Hydrogenobacter* (Ikeda et al. 2006; Yamamoto et al. 2003). The presence of the ferredoxin-dependent rTCA cycle for CO_2_ fixation indicates a possible adaptation to microaerophilic conditions by *Nitrospira*, supported by the tendency of isolates to form aggregates (Nowka et al. 2015b; Ushiki et al. 2013) and the high abundance of uncultured representatives in the oxic-anoxic interface of biofilms (Schramm et al. 2000). Furthermore, the high degree of enrichment of comammox *Nitrospira* in a bioreactor system inoculated with activated sludge and operated under low dissolved O_2_ concentrations indicates a competitive advantage of comammox over canonical ammonia oxidizers under microaerophilic conditions (Camejo et al. 2017). These findings agree with the recruitment of different terminal oxidases by the nitrifying guilds. Canonical ammonia oxidizers, except for *Nitrosomonas eutropha*, rely on the low-affinity cytochrome *aa*_3_ oxidase to transfer electrons to O_2_ (Stein et al. 2007), while *Nitrospira* use a yet biochemically uncharacterized putative cytochrome *bd*-like terminal oxidase that shows some characteristics of *cbb*_*3*_-type oxidases and might have a stronger affinity for O_2_ than the *aa*_3_-type (Lücker et al. 2010).

Taken together, genomic surveys of complete nitrifiers suggest several metabolic differences between the nitrifying guilds that potentially shape microbial community composition in the environment. In addition, the observed high ammonia affinity and growth yield of the first comammox pure culture *N. inopinata* suggest an adaptation to slow growth in highly oligotrophic habitats (Kits et al. 2017). Identification of such niche-defining factors is of global interest considering the guild-specific differences in producing N_2_O, a greenhouse gas with a 300 times higher global warming potential than CO_2_ (IPCC 2013). Canonical AOB produce N_2_O during ammonia oxidation as byproduct of hydroxylamine oxidation and in hypoxic conditions as product of nitrifier denitrification (Arp and Stein 2003; Stein 2011). In contrast, AOA produce lower amounts of N_2_O, which is mainly generated by abiotic reactions from ammonia oxidation intermediates (Kozlowski et al. 2016b; Stieglmeier et al. 2014). In agreement with these data from pure culture studies, mesocosm experiments revealed an increased N_2_O yield in fertilized soil when ammonia oxidation was dominated by AOB (Hink et al. 2018). In AOB, enzymatic N_2_O production is catalyzed by nitric oxide reductases (NOR) that convert NO derived from nitrite reduction (Kozlowski et al. 2016a), and cytochrome P460, a periplasmic metalloenzyme shown to directly convert NH_2_OH to N_2_O under anaerobic conditions (Caranto et al. 2016). Similar to AOA, the lack of NOR homologs in complete nitrifiers indicates that *Nitrospira* do not produce N_2_O via nitrifier denitrification. In addition, although some *Nitrospira* lineage II encode a protein with low similarity (< 55%) to cytochrome P460 of *N. europaea*, a homologous gene is absent in most comammox genomes. Future physiological studies focusing on potential N_2_O production of complete nitrifiers are needed to determine their potential contribution to N_2_O emissions.

## Differences in nitrogen acquisition and assimilation in *Nitrospira*

**Fig. 3:**
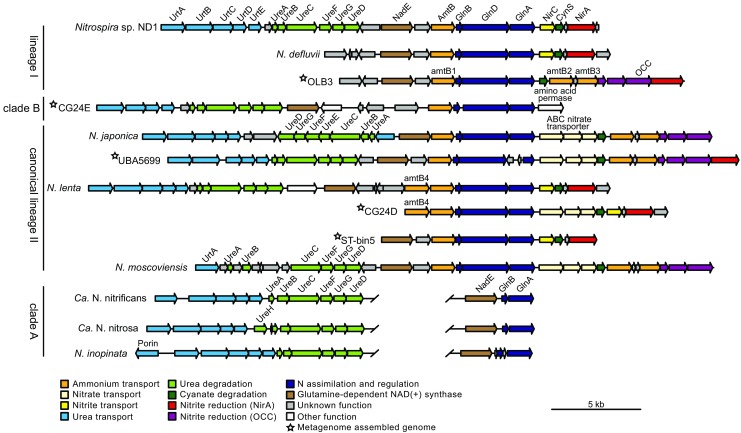
Schematic illustration of the *Nitrospira* syntenic genomic regions containing genes for N uptake and assimilation. Inferred protein functions are indicated by color, genes are drawn to scale. The scale bar corresponds to 5 kb sequence length. Affiliation with the main *Nitrospira* lineages or the comammox clades (both part of *Nitrospira* lineage II) is given on the left-hand side. AmtB, AmtB-type ammonium transporter; CynS, cyanase; OCC, octaheme cytochrome *c*; NirA, ferredoxin-dependent nitrite reductase; GlnA, glutamine synthetase; GlnB, nitrogen regulatory protein PII; GlnD, protein PII uridylyltransferase; NadE, glutamine-dependent NAD(+) synthase; NirC, nitrite transporter; UreABC, urease; UrtABCDE, urea ABC-type transporter; UreEFGD, urease accessory proteins

The general metabolic profiles of strict nitrite-oxidizing and comammox *Nitrospira* are similar, indicated by the low amount of comammox-specific genes detected by comparative genomics (Palomo et al. 2018). Features unique to comammox *Nitrospira* appear to be mainly the ammonia and hydroxylamine oxidation machinery and the apparent absence of nitrite assimilation and cyanate degradation (Palomo et al. 2018). The core genome of all analyzed *Nitrospira* includes the genes for the nitrite oxidation pathway, all five complexes of the respiratory chain, the reductive and oxidative TCA cycle, gluconeogenesis, and the pentose phosphate cycle. Interestingly, although the nitrite oxidoreductase (NXR), the enzyme catalyzing nitrite oxidation, is conserved and highly similar in all *Nitrospira* genomes, the nitrite affinity of *N. inopinata* is around 50-fold lower than for canonical *Nitrospira* (Fig. 2; Kits et al. 2017). As mentioned above, canonical and comammox *Nitrospira* also differ in their ability to use nitrite as N source. While canonical *Nitrospira* can grow under nitrite only conditions, all cultured complete nitrifiers show no growth with nitrite as sole substrate without an additional N source, which can be explained by the lack of assimilatory nitrite reductases in all available comammox genomes (Table S1; Daims et al. 2015; Palomo et al. 2018; van Kessel et al. 2015). In strict nitrite-oxidizing *Nitrospira*, genes encoding the different assimilatory nitrite reductases are co-localized with genes involved in other N acquisition and assimilation pathways (Fig. 3). Although these gene clusters differ in composition, most metabolic functions are conserved in these syntenic genome regions of canonical *Nitrospira*. Besides assimilatory nitrite reduction, the conserved metabolic functions include ammonia transport and assimilation, as well as cyanate degradation and in most genomes urea hydrolysis. Until now, two different types of assimilatory nitrite-reducing enzymes have been described for canonical *Nitrospira*: (i) the assimilatory ferredoxin-dependent nitrite reductase NirA and (ii) an octaheme cytochrome *c* (OCC) that potentially reduces nitrite to ammonia for assimilation (Koch et al. 2015; Lücker et al. 2010; Ushiki et al. 2018). The OCC of *Nitrospira* belongs to the multiheme cytochrome *c* family that harbors a variety of N-transforming enzymes, including HAO, hydrazine dehydrogenase, as well as dissimilatory penta- and octaheme nitrite reductases (Klotz et al. 2008). Although the OCC of *Nitrospira* lacks biochemical characterization, genomic context and gene expression analyses suggest a role in assimilatory nitrite reduction (Koch et al. 2015; Ushiki et al. 2018). In *Nitrospira* genomes, the gene for OCC is co-localized with two genes encoding a transmembrane Rieske/cytochrome *b* complex (Fig. 3). The similarity of these subunits to complex III of the respiratory chain suggests a direct interaction of the nitrite reductase with the quinone pool. Consistent with the supposed periplasmic localization of the OCC is the lack of the nitrite transporter NirC in *N. moscoviensis* and *N. japonica*, which both contain OCC instead of the cytoplasmic NirA. In all *Nitrospira* genomes harboring *nirA*, *nirC* is commonly located upstream of the gene for cyanate degradation, indicting a possible involvement in cyanate transport also. Intriguingly, one MAG classified as clade B comammox (bin CG24E) harbors a genomic region syntenic to this N metabolism gene cluster of canonical *Nitrospira* (Fig. 3). Although this region lacks genes for assimilatory nitrite reduction, it is tempting to speculate that other complete nitrifiers might possess the complete gene cluster and thus are able to use nitrite as N source when ammonium is temporarily not available.

**Fig. 4:**
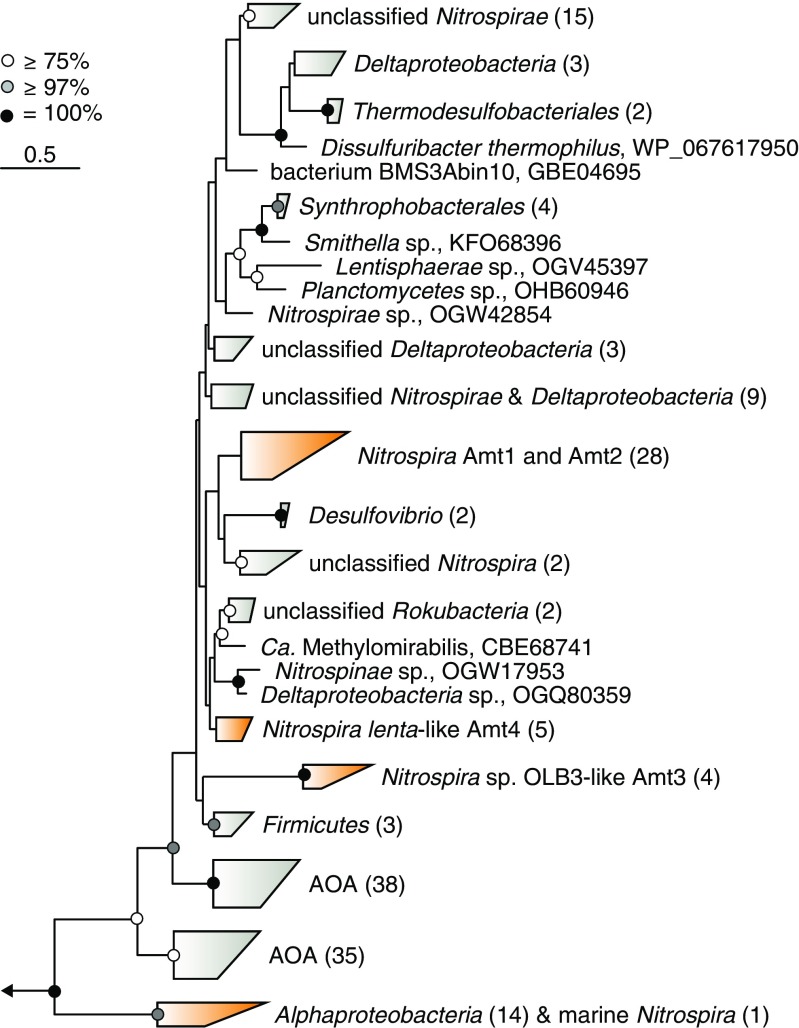
Phylogeny of *Nitrospira* ammonium transporters. The maximum likelihood tree is based on a manually refined muscle (Edgar 2004) protein alignment and was calculated with RaxML (Stamatakis 2014) on the CIPRES Science Gateway (Miller et al. 2010) using the WAG substitution and PROTCAT rate heterogeneity models and 100 bootstrap replicates. A 10% conservation filter was used, resulting in 457 alignment positions. Rh-type transporters of comammox *Nitrospira* and selected reference organisms were used to root the tree. The arrow indicates the position of the outgroup. The scale bar corresponds to 50% estimated sequence divergence. Numbers in brackets reflect the number of sequences contained in the respective sequence group

Since ammonium, in contrast to the uncharged ammonia, cannot diffuse passively through biological membranes, external ammonium has to be actively transported into the cell for N assimilation. This uptake is facilitated by members of the Amt/MEP/Rh transporter family, which are found in all domains of life. Amt-type transporters have been identified in many bacterial and archaeal groups (including AOA) and have been intensively studied in *E. coli* and *Archaeoglobus fulgidus*. In contrast, Rh-type transporters are scarce in bacteria, but interestingly found in most AOB (Offre et al. 2014). While Rh- and Amt-type transporters often co-occur in eukaryotic genomes, such co-occurrence has been rarely identified in bacterial genomes. Among the few exceptions are anaerobic ammonia oxidizers (anammox), which possess both types of transporters (Matassi 2017). Crystal structure analyses revealed that Amt- as well as Rh-type transporters form homotrimers with a central, hydrophobic pore (Khademi et al. 2004; Lupo et al. 2007; Zheng et al. 2004). Additionally, the Rh-type transporter of *N. europaea* shows a lower ammonium affinity compared to Amt-type transporters (Lupo et al. 2007; Weidinger et al. 2007). However, several other key characteristics of the ammonium transporters in nitrifiers, like substrate specificity and substrate recruitment and conduction, are still under debate (for reviews see Neuhauser et al. 2014; Offre et al. 2014). So far, all comammox clade A members possess Rh-type ammonium transporters similar (~76% amino acid identity) to betaproteobacterial AOB. In contrast, canonical and comammox clade B *Nitrospira* employ Amt-type transporters (Palomo et al. 2018). Besides the different ammonium transporter types, additional factors differentiate the comammox clades in their genetic makeup regarding ammonium uptake, including the amount and genomic localization of genes encoding for Rh/Amt transporters. Interestingly, several clade B and canonical *Nitrospira* species (Fig. 3) as well as AOA encode more than one Amt-type transporter (Koch et al. 2015; Offre et al. 2014; Palomo et al. 2018). The two Amt transporters of the marine AOA *N. maritimus* share only 39% amino acid identity and belong to different Amt-transporter clades as shown by phylogenetic analysis (Offre et al. 2014), which may indicate distinct metabolic functions. Indeed, transcriptional analysis revealed a differential expression pattern of the corresponding *amtB* genes in response to changes in ammonium availability (Qin et al. 2018). This potential functional differentiation of Amt transporters might be beneficial in environments with fluctuating ammonium concentrations. Intriguingly, some canonical *Nitrospira* encode three Amt homologs, which cluster separately in phylogenetic analyses (Fig. 4) and where the third copy shows only limited similarity (< 50%) to the other Amt transporters present in the genomes.

Besides the uptake of external ammonium, *Nitrospira* can intracellularly generate ammonia from cyanate and/or urea degradation. While the known canonical *Nitrospira* possess a cyanase for decomposing cyanate into ammonia and CO_2_, all known complete nitrifiers seem to have lost this enzyme. Similarly, most canonical ammonia oxidizers lack the genetic repertoire for cyanate degradation except for the freshwater AOA *Nitrososphaera gargensis* (Palatinszky et al. 2015). In most microorganisms, cyanate degradation is performed to either detoxify cyanate or utilize it as N source (Kamennaya et al. 2008; Luque-Almagro et al. 2008). Contrastingly, *N. gargensis* has been shown to proliferate on cyanate as sole energy substrate, and *N. moscoviensis* can provide AOB with cyanate-derived ammonia resulting in the stoichiometric conversion of cyanate to nitrate (Palatinszky et al. 2015). Cyanate is intracellularly formed during degradation of carbamoyl phosphate, an intermediate of arginine and pyrimidine biosynthesis and the urea cycle, and from thiocyanate, a common pollutant released by gold mining and other industrial processes (Allen and Jones 1964; Stratford et al. 1994). In addition, environmental cyanate sources include abiotic urea decomposition and photoproduction (Dirnhuber and Schutz 1948; Widner et al. 2016). Since it is technically challenging to quantify cyanate in environmental samples, studies analyzing its distribution in natural systems are rare. However, recent surveys indicate that cyanate, together with urea and amino acids, might be an important dissolved organic N compound in marine systems (Widner et al. 2013; Widner et al. 2016). In this context, metatranscriptomic analysis of an uncultured *Nitrospira* associated with a marine sponge revealed high expression of the cyanase encoding gene, indicating cyanate degradation by *Nitrospira* in this sponge-microbe symbiosis (Moitinho-Silva et al. 2017).

As mentioned above, urea can also be used by nitrifiers. It is enzymatically hydrolyzed to ammonia and CO_2_, and many canonical ammonia oxidizers and complete nitrifiers can use this ammonia as energy and N source (Alonso-Saez et al. 2012; Daims et al. 2015; Lu and Jia 2013; Pommerening-Röser and Koops 2005; van Kessel et al. 2015). Canonical ureolytic *Nitrospira* can degrade urea for N assimilation and, additionally, provide ammonia to non-ureolytic ammonia oxidizers, thus initiating full nitrification in a reciprocal feeding interaction (Koch et al. 2015). The genetic inventory for urea hydrolysis has been identified in many comammox and canonical *Nitrospira* (Table S1). It includes a nickel (Ni)-dependent urease (UreABC) as well as accessory proteins (UreDFG) for the maturation of the holoenzyme (Farrugia et al. 2013). Additionally, all urease-positive *Nitrospira* isolates except for *N. moscoviensis* possess a complete gene set for an ATP-dependent ABC-type urea transporter (UrtABCDE) encoded upstream of the urease structural genes (Fig. 3). This type of transporter is characterized by its high affinity for urea (Valladares et al. 2002), indicating an adaptation to low urea concentrations in the environment. In contrast to strict nitrite-oxidizing *Nitrospira*, complete nitrifiers employ two additional urea transporters, a urea carboxylase-related transporter and an outer-membrane porin (Palomo et al. 2018). Phylogenetic analysis of the urease gamma subunit (UreA) revealed a close affiliation of most *Nitrospira* UreA, except for *N. japonica* (Ushiki et al. 2018). Aside from the distinct UreA, the urease cluster of *N. japonica* possesses other species-specific features, including genes encoding the metallo-chaperone UreE and an additional urea permease, which forms an urea channel for diffusion through the membrane in a pH independent manner (Sebbane et al. 2002). The Ni-chaperone UreE is supposed to insert nickel into the urease apoprotein (Farrugia et al. 2013). For other ureolytic *Nitrospira*, it has been hypothesized that the (NiFe)-hydrogenase maturation enzymes HypA and HypB compensate the lack of UreE (Koch et al. 2015). This distinct urease operon of *N. japonica* underlines the genomic flexibility of *Nitrospira* especially in this particular genomic region (Fig. 3). Here, comparable to the different assimilatory nitrite reductases (see above), also the urease functional modules have been exchanged in different *Nitrospira* species. In addition to comammox and canonical lineage II *Nitrospira*, ureolytic activity was also observed in *Nitrospira* sp. ND1, a lineage I *Nitrospira* isolated from activated sludge (Ushiki et al. 2018). Together with the identification of a urease operon in a sponge-associated lineage IV *Nitrospira* genome bin (Slaby et al. 2017), this shows a broad distribution of the urea hydrolyzing capability within the genus *Nitrospira*.

## Metabolic versatility of *Nitrospira*

Members of the genus *Nitrospira* were considered to be of restricted metabolic capability and their presence in the environment was thus used as proxy for nitrite oxidation. However, recent studies identified a much broader metabolic flexibility, including aerobic growth on formate and hydrogen (H_2_) and anaerobic reduction of nitrate to nitrite in the presence of suitable electron donors (Daims et al. 2016; Ehrich et al. 1995; Koch et al. 2014; Koch et al. 2015). Comparative genomics did not reveal a prevalence of canonical or comammox *Nitrospira* in recruiting additional metabolic capacities, and many of the alternative metabolic features are not restricted to a certain group within *Nitrospira*. Two widely distributed metabolic traits for energy conservation are formate and H_2_ oxidation. The genetic setup for formate oxidation includes genes encoding a formate transporter and the three subunits of formate dehydrogenase. This gene cluster was identified in most canonical *Nitrospira* as well as in clade B comammox (Palomo et al. 2018). The capability of oxidizing formate was confirmed under oxic and anoxic conditions for *N. moscoviensis* (Koch et al. 2015). In addition, formate incorporation by uncultured *Nitrospira* was also observed in activated sludge samples in the presence and absence of nitrite under oxic conditions (Gruber-Dorninger et al. 2015).

Besides formate, H_2_ is a common fermentation product and the capability to exploit these substrates is especially advantageous in hypoxic or anoxic habitats. Two different types of hydrogenases have been identified in *Nitrospira* genomes to date. All hydrogenases identified in comammox *Nitrospira* belong to the [NiFe]-hydrogenase group 3b, a large enzyme family with distinct physiological roles. On the one hand, these soluble cytoplasmic, bidirectional enzymes can produce H_2_ by reoxidizing NAD(P)H to maintain the cellular redox balance during fermentation (Berney et al. 2014b). On the other hand, they can provide electrons for CO_2_ fixation by oxidizing H_2_ in *Hydrogenobacter* (Yoon et al. 1996), as was recently also hypothesized for mixotrophic verrucomicrobial methanotrophs (Carere et al. 2017). Furthermore, these hydrogenases might also be involved in sulfur cycling by reducing elemental sulfur or polysulfide to H_2_S as shown for hyperthermophilic archaea (Ma et al. 2000). However, the detection of H_2_S production by *Mycobacterium smegmatis* under O_2_ limitation in a mutant lacking all hydrogenases challenges the contribution of group 3b hydrogenase to this process, at least in this bacterium (Berney et al. 2014a). The metabolic function of this type of hydrogenase in complete nitrifiers remains to be determined and might include (i) H_2_ oxidation as alternative or additional electron source for energy conservation and/or CO_2_ fixation, (ii) H_2_ evolution for maintaining the redox balance during anaerobic degradation of simple organic matter (as suggested by Kits et al. 2017), and (iii) H_2_S production for sulfur assimilation. Moreover, the identification of group 3b hydrogenases in canonical ammonia and nitrite oxidizer genomes, like the marine NOB *Nitrococcus mobilis* (Füssel et al. 2017), *Nitrospina marina* (Lücker et al. 2013), and several AOB including *Nitrosococcus halophilus* Nc4 (GenBank accession number ADE14678.1) and *Nitrosomonas mobilis* (Thandar et al. 2016) emphasizes the need to characterize the physiological function(s) of this enzyme in nitrifying bacteria. *N. moscoviensis* is the only *Nitrospira* species known to have recruited a different type of hydrogenase. In this organism, a cytoplasmic group 2a [NiFe]-hydrogenase enables aerobic growth with H_2_ as sole substrate (Koch et al. 2014). Notably, nitrite and H_2_ can be oxidized simultaneously, indicating metabolic compatibility of these substrates and a lack of substrate preference. Furthermore, *N. moscoviensis* can couple H_2_ oxidation to anaerobic nitrate reduction (Ehrich et al. 1995).

The lack of known dissimilatory nitrate reductases in the genome of *N. moscoviensis* points to a reversibility of NXR. Contrastingly, in *N. inopinata* a putative periplasmic nitrate reductase (NAP) might additionally catalyze nitrate reduction in the presence of suitable electron donors (Kits et al. 2017). Intriguingly, some *Nitrospira* genomes furthermore contain a pentaheme nitrite reductase (NrfAH), including *N. inopinata* and *Nitrospira* sp. ND1 (Table S1). NrfAH catalyzes dissimilatory nitrite reduction to ammonium (DNRA) during anaerobic growth on low-potential electron donors. This metabolic capability suggests an additional ecological function of *Nitrospira* in the biogeochemical N cycle. While complete nitrifiers represent an ammonia sink, *Nitrospira* performing DNRA would produce ammonia and act as source of this N compound. The observed metabolic versatility of *Nitrospira* may be essential for successful adaptation to fluctuating environmental conditions. However, this flexibility poses a challenge when inferring the function of *Nitrospira* in the environment, since their occurrence and abundance might not correlate with nitrification activity.

## Isolation of comammox *Nitrospira*

Genomic approaches can yield a great amount of novel insights into the metabolic potential of an organism or a complex microbial community. However, novel physiologies cannot be determined based on genome-inferred information alone. In order to facilitate the study of fastidious microorganisms like nitrifiers, state-of-the-art cultivation-independent methods have successfully been employed to study comammox and canonical *Nitrospira* in enrichment cultures or directly in their environment (Daims et al. 2015; Gruber-Dorninger et al. 2015; van Kessel et al. 2015). While these approaches allow the direct confirmation of proposed physiologies on single-cell level, they cannot completely replace classical cultivation-dependent physiological characterizations, mainly due to their dependency on specialized equipment and laboratory setups (reviewed by Singer et al. 2017) and potential metabolic interactions with co-occurring microbes. However, the isolation of novel complete nitrifiers is challenging due to their low growth rates and the difficulty to separate them from other nitrifying and heterotrophic microbes. Until now, *N. inopinata* is the only available comammox pure culture (Kits et al. 2017). This complete nitrifier was first highly enriched in batch cultures that were regularly transferred (Daims et al. 2015). Subsequently, a pure culture was obtained by dilution to extinction (Kits et al. 2017). In contrast to this classical cultivation approach, a co-enrichment of *Ca*. N. nitrosa and *Ca*. N. nitrificans was obtained in a hypoxic bioreactor system operated in sequencing-batch mode and supplied with low concentrations of ammonium, nitrite, and nitrate (van Kessel et al. 2015). Like most nitrifying microorganisms, the vast majority of known *Nitrospira* species prefers to grow in dense microcolonies in biofilm-like structures (Nowka et al. 2015b; Ushiki et al. 2013). This makes it virtually impossible to separate them from accompanying heterotrophic contaminants by classical cultivation methods alone. To circumvent this challenge, physical isolation methods can be employed to segregate *Nitrospira* cells or clonal microcolonies from their heterotrophic companions in pre-enriched cultures. These include the use of label-free cell sorting (Fujitani et al. 2014), optical tweezers (Nowka et al. 2015b), and a combination of Raman microspectroscopy and microfluidic cell sorting (reviewed in Huys and Raes 2018). These techniques also hold the biggest promise to obtain pure cultures of comammox *Nitrospira* derived from engineered environments like drinking and wastewater treatment systems, which will be invaluable to study their role and competitive niche, and to elucidate their biotechnological potential in order to optimize sustainable water treatment in the future.

## Conclusion

Over the last years, our understanding of nitrification and nitrifying microorganisms dramatically improved due to the identification of novel key players, like AOA and complete nitrifiers. Furthermore, the identification of novel metabolic pathways and interactions, like the potential reciprocal feeding interactions of aerobic nitrifiers based on urea and cyanate hydrolysis, revolutionized our view of the N cycle. These milestones in nitrification research show that aerobic ammonia oxidation to nitrate is much more complex than simple cross-feeding between two functional groups. The recent identification of the long-sought complete nitrifiers within the genus *Nitrospira* not only overturned a century-old dogma of nitrification research; it also demonstrated the questionability of simplified correlations of metabolic functions to taxonomy-defined groups. Without in situ activity determination and/or a combination of genomic and transcriptomic data, it is difficult to assign a metabolic function to *Nitrospira* in the environment, since members of this genus could perform full nitrification, nitrite oxidation, or other alternative lifestyles beyond the N cycle-like formate or hydrogen oxidation. Thus, more targeted approaches to identify and isolate comammox *Nitrospira* are needed to reveal and confirm niche-separating physiological features and to further assess the ecological significance of complete nitrification in natural and engineered ecosystems.

## Electronic supplementary material

ESM 1 (PDF 1.20 MB)
