# Supplementary material for: Complete nitrification: insights into the ecophysiology of comammox Nitrospira
Source: Appl Microbiol Biotechnol. 2018 Nov 10;103(1):177–89. doi: 10.1007/s00253-018-9486-3 (PMC6311188; doi:10.1007/s00253-018-9486-3)
Supplement: Supplementary file 1 — (PDF 1.20 MB) [file 253_2018_9486_MOESM1_ESM.pdf]

Supplementary material

Journal:

Applied Microbiology and Biotechnology

Title:

Complete nitrification: insights into the ecophysiology of comammox Nitrospira

Authors:
Hanna Koch, Maartje A. H. J. van Kessel, Sebastian Lücker*

Affiliation:
Department of Microbiology, Radboud University Nijmegen, The Netherlands

*address correspondence to: Sebastian Lücker, s.luecker@science.ru.nl

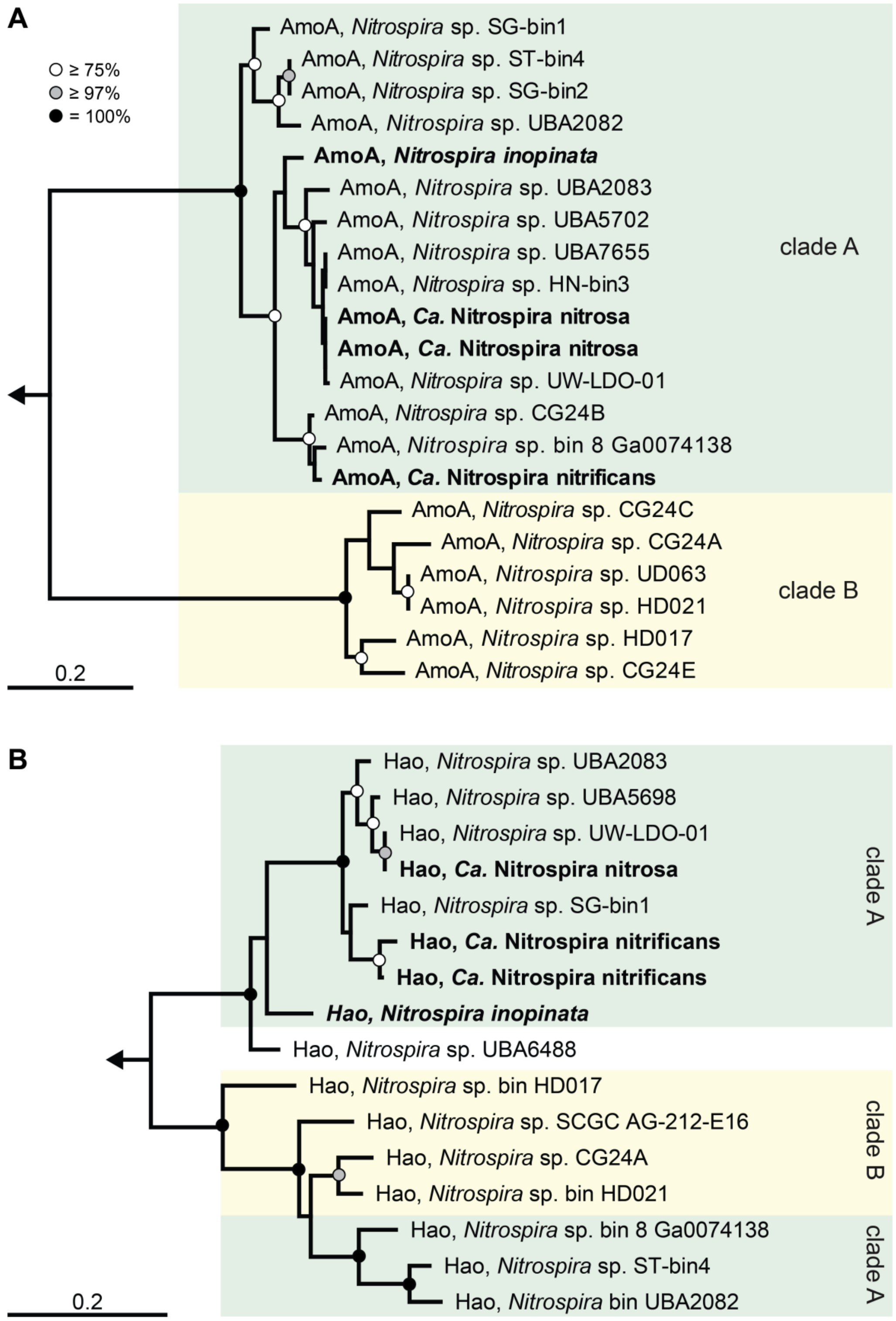

Figure S1: Comparison of **(A)** AmoA and **(B)** HaoA-based phylogeny of comammox *Nitrospira*. Protein sequences were aligned using Muscle (Edgar 2004) and maximum likelihood trees were calculated in Geneious (version 11.1.3) using PhyML (Guindon et al. 2009) with the WAG substitution model and 100 bootstrap iterations. Betaproteobacterial protein sequences were used to root the trees. The arrow indicates the position of the outgroup.

Table S1: Summary of key features of publicly available *Nitrospira* genomes in the NCBI database.

Strain	ccmABCDEFGHI[a] (N.inopinata)[b]	HAO (N.inopinata)	CycAB (N.inopinata)	AmoABC (N.inopinata)	NxrABC (N.inopinata)	NrfAH (N.inopinata)	NapA (N.inopinata)	NirK (N.inopinata)	Rh-type AMT (N.inopinata)	AmtB- type AMT (N.moscoviensis)	UreABCDFG (N.inopinata)	UrtABCDE (N.inopinata)	NirA (N.lenta)	OCC (N.moscoviensis)	NirC (N.lenta)	NrtABD (N.moscoviensis)	Cyanase (N.moscoviensis)	Formate dehydrogenase (N.moscoviensis)	3b [NiFe] hydrogenase large subunit (N.inopinata)	Comammox clade	Nitrospira lineage	Scaffolds[c]	Enviromental source[e]	CheckM - completeness	Reference
N. sp. CG24A	ABCDEFGHI	AB	AB	ABC	BC			yes		yes		ABCDE						AB focA[d]		B	2	58	DWTP	87,22	Palomo et al. 2018
N. sp. CG24B	ABCDEFGHI	AB	AB	ABC	BC			yes	yes		ABCDFG	AB								A	2	36	DWTP	92,22	Palomo et al. 2018
N. sp. CG24C	ABCDEFGHI			ABC	BC			yes		yes	ABCDFG	A						AB focA		B	2	28	DWTP	93,05	Palomo et al. 2018
N. sp. CG24D	BCDEFGHI				ABC			yes		yes			yes		yes	ABD	yes	AB focA			2	135	DWTP	92,17	Palomo et al. 2018
N. sp. CG24E	ABFGHI			AB	ABC					yes	ABCFG	BCDE						ABG focA		B	2	63	DWTP	93,05	Palomo et al. 2018
N. sp. HD017	ABCDEFGHI	AB	AB	ABC	C					yes	ABCFG	ABD								B	2	589	soil	84,04	Orellana et al. 2018
N. sp. HD021	ABCDEFGHI	AB	AB	ABC	ABC			yes		yes	ABCDFG	ABCD						A		B	2	627	soil	89,56	Orellana et al. 2018
N. sp. HN-bin3	CDEGHI	B	AB	AB	BC				yes		ABCG								yes	A	2	357	tap water China	78,22	Wang et al. 2017
**N. defluvii**					ABC			yes		yes			yes		yes		yes	AB focA			1	**closed**	WWTP	97,67	Lücker et al. 2010
**N. inopinata**	ABCDEFGHI	AB	AB	ABC	ABC	AH	yes	yes	yes		ABCDFG	ABCDE							yes	A	2	**closed**	pipe biofilm	96,82	Daims et al. 2015
**N. moscoviensis**					ABC	H		yes		yes	ABCDFG	A		yes		ABD	yes	ABG focA			2	**closed**	pipe heating system	95,91	Koch et al. 2015
**Ca. N. nitrificans**	ABCDEFGHI	AB	AB	ABC	ABC			yes	yes		ABCDFG	ABCDE							yes	A	2	36	trickling filter aquaculture	96,76	van Kessel et al. 2015
**Ca. N. nitrosa**	ABCDEFGHI	AB	AB	ABC	ABC			yes	yes		ABCDFG	ABCDE							yes	A	2	15	trickling filter aquaculture	96,76	van Kessel et al. 2015
**N . japonica**					ABC			yes		yes	ABCG	ABCDE		yes		ABD	yes				2	**closed**	WWTP	96,82	Ushiki et al. 2018
**N. sp. ND1**					ABC	AH		yes		yes	ABCDFG	ABCDE	yes		yes		yes	AB focA			1	6	WWTP	97,67	Ushiki et al. 2018
N. sp. OLB3					ABC			yes		yes			yes	yes			yes	ABG			1	79	WWTP	91	Speth et al. 2016
N. sp. SCGC	ABCEFGHI	AB	AB	BC	ABC			yes										A focA			2	162	single cell; soil	52,67	GenBank: LSTC01000001.1
N. sp. bin75					ABC					yes	ABCG	ABCDE	yes		yes	ABD	yes				4	115	marine sponge	94,09	Slaby et al. 2017
N. sp. SCN59-13					C			yes		yes	DFG							AB focA			1	212	bioreactor	35,96	Kantor et al. 2015
N. sp. bin8	ABCDEFGHI	AB	AB	AB	AC			yes	yes		ABCDFG	ABCDE							yes	A	2	61	DWTP	87,67	Pinto et al. 2015
N. sp SG-bin1	ABCDEFGHI	AB	AB	ABC	ABC			yes	yes		ABCDFG								yes	A	2	48	tap water Singapore	95,85	Wang et al. 2017
N. sp SG-bin2	ABCEFGHI			AB	ABC			yes	yes		ABCDFG	ABDE							yes	A	2	63	tap water Singapore	95,85	Wang et al. 2017
N. sp ST-bin4	ABCDEFGHI	A	AB	AB	AC			yes	yes		ABCDFG	DE							yes	A	2	117	tap water USA	92,06	Wang et al. 2017
N. sp ST-bin5					ABC	A		yes		yes			yes		yes		yes	AB focA			2	29	tap water USA	91,82	Wang et al. 2017
N. sp. UBA2082	ABCDEFGHI	A	AB	ABC	ABC			yes	yes		ABCDFG	ABCD							yes	A	2	37	residential sediments after rain	93,12	Parks et al. 2017
N. sp. UBA2083	AFGHI	AB	AB	AB	BC			yes	yes		ABCDFG									A	2	137	residential sediments after rain	87,32	Parks et al. 2017
N. sp. UBA4129					AC			yes		yes					yes		yes	A focA			1	376	WWTP	80,75	Parks et al. 2017
N. sp. UBA5698	ABCDEFGHI	AB	AB		ABC			yes	yes		ABCDFG	ABCD							yes	A	2	73	industrial sediments after rain	95	Parks et al. 2017
N. sp. UBA5699	BCDEFGI				C			yes		yes	ABCDFG	ABCDE	yes	yes		ABD	yes				2	124	industrial sediments after rain	90,3	Parks et al. 2017
N. sp. UBA5702	ABCDEGHI		AB	AB	ABC			yes	yes		ABCDFG	ABCDE							yes	A	2	109	industrial sediments after rain	94,09	Parks et al. 2017
N. sp. UBA6488	BCDEFGHI	A			BC			yes		yes		A	yes		yes		yes		yes		2	95	river estuary	82,23	Parks et al. 2017
N. sp. UBA6493	BCDEFGHI				C			yes		yes			yes		yes		yes		yes		2	52	river estuary	81,58	Parks et al. 2017
N. sp. UBA667					BC	AH		yes		yes								B focA			1	376	WWTP	76,94	Parks et al. 2017
N. sp. UBA6909	ABCDEFGHI	AB	AB		ABC		yes		yes		ABCDFG	A									2	275	river estuary	86,53	Parks et al. 2017
N. sp. UBA7240					C	AH		yes					yes				yes	AB focA			1	412	WWTP (anoxic tank)	69,12	Parks et al. 2017
N. sp. UBA7655	ABCDEFGHI		B	AB	ABC			yes	yes		ABCDFG								yes	A	2	132	DWTP	78,07	Parks et al. 2017
N. sp. UD063	ABEH		AB	ABC	BC			yes		yes	ABCDG	ABCD						AB focA		B	2	1453	soil	91,16	Orellana et al. 2018
N. sp. UW-LDO-01	ABCDEFGHI	AB	AB	AB	AC			yes	yes		ABCDFG	ABCDE							yes	A	2	230	lab-scale bioreactor	95,8	Camejo et al. 2017
N. sp. UW-LDO-02					AC	AH		yes		yes			yes		yes		yes	AB focA			1	522	lab-scale bioreactor	94,36	Camejo et al. 2017

[a] AmoABC, ammonia monooxygenase; AMT, ammonium transporter; CCM, cytochrome c-biosynthesis proteins; CycAB: cytochromes c554 and cM552; HAO, hydroxylamine dehydrogenase; Nap, periplasmic nitrate reductase; NirA, ferredoxin-dependent nitrite reductase; NirC, nitrite transporter; NirK, copper-dependent nitrite reductase; NrfAH, pentaheme nitrite reductase; NrtABC, nitrate ABC-type transporter; Nxr, nitrite oxidoreductase; OCC, octaheme cytochrome c; UreABCDFG, urease and urease accessory proteins; UrtABCDE, urea ABC-type transporter.

[b] Homologous proteins were identified using BLASTp (Altschul et al. 1990). Only hits with >50% identity and >50% query coverage were considered as homologs. *Nitrospira* species used as query are indicated in brackets.

[c] number of scaffolds according to NCBI

[d] focA: Formate transporter

[e] DWTP, drinking water treatment plant; WWTP, wastewater treatment plant

References

Altschul SF, Gish W, Miller W, Myers EW, Lipman DJ (1990) Basic local alignment search tool. J Mol Biol 215(3):403–410. https://doi.org/10.1016/S0022-2836(05)80360-2

Camejo PY, Domingo JS, McMahon KD, Noguera DR (2017) Genome-enabled insights into the ecophysiology of the comammox bacterium "*Candidatus* Nitrospira nitrosa". mSystems 2(5):16. https://doi.org/10.1128/mSystems.00059-17

Daims H, Lebedeva EV, Pjevac P, Han P, Herbold C, Albertsen M, Jehmlich N, Palatinszky M, Vierheilig J, Bulaev A, Kirkegaard RH, von Bergen M, Rattei T, Bendinger B, Nielsen PH, Wagner M (2015) Complete nitrification by *Nitrospira* bacteria. Nature 528(7583):504–509. https://doi.org/10.1038/nature16461

Edgar RC (2004) MUSCLE: multiple sequence alignment with high accuracy and high throughput. Nucleic Acids Res 32(5):1792–1797. https://doi.org/10.1093/nar/gkh340

Guindon S, Delsuc F, Dufayard J-F, Gascuel O (2009) Estimating maximum likelihood phylogenies with PhyML. In: Posada D (ed) Bioinformatics for DNA sequence analysis. Humana Press, Totowa, NJ, pp 113–137

Kantor RS, van Zyl AW, van Hille RP, Thomas BC, Harrison STL, Banfield JF (2015) Bioreactor microbial ecosystems for thiocyanate and cyanide degradation unravelled with genome-resolved metagenomics. Environ Microbiol 17(12):4929–4941. https://doi.org/10.1111/1462-2920.12936

Koch H, Lücker S, Albertsen M, Kitzinger K, Herbold C, Spieck E, Nielsen PH, Wagner M, Daims H (2015) Expanded metabolic versatility of ubiquitous nitrite-oxidizing bacteria from the genus *Nitrospira*. Proc Natl Acad Sci U S A 112(36):11371–11376. https://doi.org/10.1073/pnas.1506533112

Lücker S, Wagner M, Maixner F, Pelletier E, Koch H, Vacherie B, Rattei T, Damste JSS, Spieck E, Le Paslier D, Daims H (2010) A *Nitrospira* metagenome illuminates the physiology and evolution of globally important nitrite-oxidizing bacteria. Proc Natl Acad Sci U S A 107(30):13479–13484. https://doi.org/10.1073/pnas.1003860107

Orellana LH, Chee-Sanford JC, Sanford RA, Loffler FE, Konstantinidis KT (2018) Year-round shotgun metagenomes reveal stable microbial communities in agricultural soils and novel ammonia oxidizers responding to fertilization. Appl Environ Microbiol 84(2):14. https://doi.org/10.1128/aem.01646-17

Palomo A, Pedersen AG, Fowler SJ, Dechesne A, Sicheritz-Pontén T, Smets BF (2018) Comparative genomics sheds light on niche differentiation and the evolutionary history of comammox *Nitrospira*. Isme J 12(7):1779–1793. https://doi.org/10.1038/s41396-018-0083-3

Parks DH, Rinke C, Chuvochina M, Chaumeil PA, Woodcroft BJ, Evans PN, Hugenholtz P, Tyson GW (2017) Recovery of nearly 8,000 metagenome-assembled genomes substantially expands the tree of life. Nat Microbiol 2(11):1533–1542. https://doi.org/10.1038/s41564-017-0012-7

Pinto AM, Marcus DN, Ijaz UZ, Bautista-de lose Santos QMB, Dick GJ, Raskin L (2015) Metagenomic evidence for the presence of comammox *Nitrospira*-like bacteria in a drinking water system. mSphere 1(1):e00054-15. https://doi.org/10.1128/mSphere.00054-15

Slaby BM, Hackl T, Horn H, Bayer K, Hentschel U (2017) Metagenomic binning of a marine sponge microbiome reveals unity in defense but metabolic specialization. Isme J 11(11):2465–2478. https://doi.org/10.1038/ismej.2017.101

Speth DR, in 't Zandt MH, Guerrero-Cruz S, Dutilh BE, Jetten MSM (2016) Genome-based microbial ecology of anammox granules in a full-scale wastewater treatment system. Nat Commun 7:10. https://doi.org/10.1038/ncomms11172

Ushiki N, Fujitani H, Shimada Y, Morohoshi T, Sekiguchi Y, Tsuneda S (2018) Genomic analysis of two phylogenetically distinct *Nitrospira* species reveals their genomic plasticity and functional diversity. Front Microbiol 8(2637). https://doi.org/10.3389/fmicb.2017.02637

van Kessel MAHJ, Speth DR, Albertsen M, Nielsen PH, Op den Camp HJM, Kartal B, Jetten MSM, Lücker S (2015) Complete nitrification by a single microorganism. Nature 528(7583):555–559. https://doi.org/10.1038/nature16459

Wang YL, Ma LP, Mao YP, Jiang XT, Xia Y, Yu K, Li B, Zhang T (2017) Comammox in drinking water systems. Water Res 116:332–341. https://doi.org/10.1016/j.watres.2017.03.042
